# Optical control of purinergic signaling

**DOI:** 10.1007/s11302-021-09799-2

**Published:** 2021-06-22

**Authors:** Tao Wang, Henning Ulrich, Alexey Semyanov, Peter Illes, Yong Tang

**Affiliations:** 1 International Collaborative Centre On Big Science Plan for Purinergic Signalling, Chengdu University of Traditional Chinese Medicine, Chengdu, China; 2 Acupuncture and Chronobiology Key Laboratory of Sichuan Province, Chengdu, China; 3 Department of Biochemistry, Institute of Chemistry, University of São Paulo, São Paulo, Brazil; 4 Shemyakin-Ovchinnikov Institute of Bioorganic Chemistry, Russian Academy of Sciences, Moscow, Russia; 5 Sechenov First Moscow State Medical University, Moscow, Russia; 6 Rudolf Boehm Institute for Pharmacology and Toxicology, University of Leipzig, Leipzig, Germany

**Keywords:** Purinergic signaling, P1 receptors, P2X receptors, P2Y receptors, Optopharmacology, Caged compounds, Photoswitchable compounds, Optogenetics

## Abstract

Purinergic signaling plays a pivotal role in physiological processes and pathological conditions. Over the past decades, conventional pharmacological, biochemical, and molecular biology techniques have been utilized to investigate purinergic signaling cascades. However, none of them is capable of spatially and temporally manipulating purinergic signaling cascades. Currently, optical approaches, including optopharmacology and optogenetic, enable controlling purinergic signaling with low invasiveness and high spatiotemporal precision. In this mini-review, we discuss optical approaches for controlling purinergic signaling and their applications in basic and translational science.

## Introduction

**Fig. 1:**
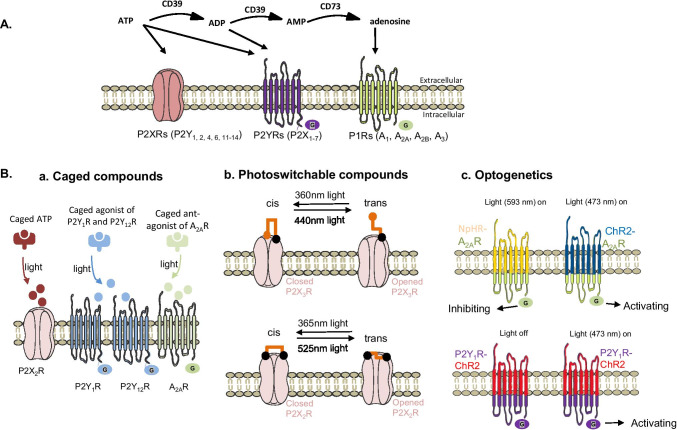
Three types of optical approaches may control purinergic signaling. **A** The concept of purinergic signaling. ATP is sequentially degraded to ADP, AMP, ADP, and adenosine by ecto-ATPase (CD39) and 5′-nucleotidase (CD73). Purinergic receptors have been classified into three types: P1Rs (A_1_, A_2A_, A_2B_, A_3_) that are only sensitive to adenosine, P2XRs (P2X_1–7_) which are selectively activated by ATP, and P2YRs (P2Y_1, 2, 4, 6, 11–14_) which are activated by both ATP, ADP, and further nucleotides. P2XRs are characterized by two transmembrane spanning regions (TM1 and TM2) and a large extracellular loop, while P2Ys and P1Rs consist of seven transmembrane spanning regions. **B** Three types of optical approaches for controlling purinergic signaling. **a** Caged compounds: Photolysis of caged ATP, caged agonist of P2Y1R and P2Y12R, and caged A_2A_R antagonist enables the rapid control of purinergic receptors by light. **b** Photoswitchable compounds: P2XRs channels can be opened or closed by introducing photoswitches to a defined site of them. **c** Optogenetics: Optogenetic control of A_2A_R and P2Y_1_R can be achieved by the introduction of genetically encoded photosensitive opsin. A total of 593 nm light in NpHR-A_2A_R enables inhibiting A_2A_R signaling while 473 nm light in ChR2-A_2A_R activates signaling of this receptor subtype. A total of 473 nm light excitation of the P2Y_1_R-ChR2 activates P2Y_1_R signaling

The concept of purinergic signaling was first proposed in 1972 when Burnstock stated that adenosine triphosphate (ATP) not only participates in the intracellular storage of energy but is also an extracellular transmitter/signaling molecule [13]. Subsequently, a range of purinergic receptors (Rs) was cloned and characterized: four types of P1Rs (G protein-coupled receptors, A_1_, A_2A_, A_2B_, A_3_) [21], seven types of P2XRs (ligand-gated cationic channels, P2X_1–7_), and eight types of P2YRs (G protein-coupled receptors, P2Y_1, 2, 4, 6, 11–14_) [1, 30, 31, 33] (Fig. 1A). Both P1Rs and P2YRs are G protein-coupled receptors and consist of seven transmembrane (TM) proteins. However, P1Rs are selectively activated by extracellular adenosine, which is obtained by dephosphorylation of its precursor entities: ATP, adenosine diphosphate (ADP), and adenosine monophosphate (AMP) [9], whereas P2YRs are activated by ATP, as well as by ADP [31]. In contrast with these G protein-coupled receptors, P2XRs are ligand-gating ion channels and characterized by two transmembrane (TM1 and TM2) proteins. P2XRs are only sensitive to ATP and undergo a conformational change in the channel upon ATP activation [30]. These purinergic receptors are widely distributed throughout the body and show great diversity in functions. If we want a high-resolution view of how individual purinergic receptor carries out specific tasks, we need high-resolution tools for controlling the activity of these receptors. In the past, many pharmacological drugs selectively targeting purinergic receptor subtypes have been developed, but they do not distinguish between the same purinergic receptors expressed in subtypes of neurons or different brain regions. Thus, the lack of tissue-specific selectivity may trigger undesirable side effects. For instance, therapeutic use of the A_2A_R agonist, regadenoson, is always associated with off-side effects, including headache, nausea, chest discomfort, or dizziness (available at http://clinicaltrials.gov). Moreover, the temporal precision of these drugs is limited by the diffusion, transport, or metabolism of active compounds. Although genetic tools enable the knock-in or knock-out of purinergic receptor subtype genes in defined subtypes of neurons or brain regions, they have inherent limitations due to the lack of spatial precision. The lack of spatiotemporal precision prevents researchers from fully understanding the role of purinergic signaling in both physiological and pathological conditions and further designing effective therapies. Therefore, novel approaches with the ability of quickly and precisely controlling purinergic signaling are needed.

Recently, optical approaches to control receptor and channel activities by light are transforming neuroscience research [35, 50, 54]. The use of light can be advantageous as light is non-invasive, can be modulated in its intensity within femtoseconds, and can be delivered in a highly controlled manner in space and time, which can overcome some of the shortcomings of conventional techniques. Two main types of optical approaches have been used for the control of purinergic signaling: optopharmacology and optogenetics.

Optopharmacology, also known as photopharmacology, refers to confer light sensitivity to a freely diffusible ligand, rather than to a target protein [50]. Since it first emerged in the 1970s when several photoreactive ligands were synthesized [7, 36], optopharmacology has boomed in neuroscience in recent years. The simplest and most widely used photosensitive chemicals are caged compounds, which are chemically modified with photoremovable protecting groups and is biologically inert before photolysis. Irradiation breaks the chemical modifications and thus results in a concentration jump of biologically active molecules in a time-dependent manner. The first attempt to synthesized caged compound dates back to 1978, when Kaplan and coworkers synthesized NPE (P3-1-(2-nitro)phenyl–ethyl), the first photoremovable protecting group, to modify ATP [32]. Subsequently, various photoremovable protecting groups, such as P3-3′,5′-dimethoxybenzoic acid (DMB) [58] and P3-[1-(4,5-dimethoxy-2-nitrophenyl)ethyl] (DMNPE) [8], have been synthesized. To date, many biomolecules or second messengers, including calcium [2], neurotransmitters [47], nucleotides [8], and peptides [39], have been caged to control cellular chemistry and physiology. However, the process of photolysis is irreversible due to the light-induced break of chemical bonds. Conversely, photoswitchable compounds enable to light-control receptors reversibly. When the target receptors are chemically attached with synthetic photoswitchable compounds, light can induce conformational changes between cis and trans isomer of the photoswitchable compounds and thereby trigger a reversible on–off control of these receptors [29, 63]. These synthetic photoswitchable compounds allow for the spatiotemporal and reversible control of a wide range of biological targets, including ion channels [6], transporters [15], G protein-coupled receptors [23], and enzymes [43], and also show huge potential in clinical treatment [19, 59].

Optogenetics is another powerful optical technique. The term optogenetics was first introduced by Deisseroth and colleagues in 2006 when they reported that the expression of microbial opsin genes in mammalian neurons resulted in the precise control of neural activity in a millisecond timescale [10]. Over the past decade, optogenetics was rapidly adopted to photoactivation and photoinhibition of cellular activities and probe neuronal functions [16]. Optogenetics strategy relies on the genetical modification of endogenous proteins with microbial opsin, including light-driven ion pumps, such as bacteriorhodopsins (BRs) and halorhodopsins (HRs), and ion channels, such as channelrhodopsins (ChRs) [55]. This contrasts with optopharmacology, in which chemical synthesis was necessary. In addition, a novel class of genetically encoded optogenetics tools, optoXRs, which are chimeric proteins coupled to different intracellular G protein–initiated signaling cascades, has been developed for selective control of Gs and Gq signaling [56].

Here, we provide a review which summarizes the applications of optical approaches in controlling purinergic signaling and their applications in investigating purinergic signaling and also discuss important considerations when applying to manipulate purinergic signaling.

## Optopharmacology for controlling purinergic signaling

In the past few years, chemists have developed various photosensitive drugs for the control of purinergic receptors, including caged compounds and photoswitchable compounds. Compared to conventional agonists or antagonists, such photochemicals offer great temporal and spatial precision. First, fast photolysis of caged compounds or light switching by photoswitchable compounds allows the control of purinergic receptors at a millisecond timescale, which is consistent with the temporal dynamics of endogenous cellular activity. Second, light delivered by the illumination device can be focused onto targeted areas of interest. Therefore, the spatiotemporal control of purinergic receptors by photosensitive chemicals permits a real-time link between the activity of purinergic receptors and a defined biological or physiological response in cells or living organisms.

## Caged compounds

Caged ATP is widely utilized to control the activation of purinergic receptors (Fig. 1a). When added to the bath with a micropipette, caged ATP is biologically inert with the absence of light stimulation while it could produce free ATP within milliseconds [20]. With this strategy, Zemelman and coworkers found that photostimulation (26 mW∙mm^−2^ of optical power at wavelengths < 400 nm) of DMNPE-caged ATP could quickly activate heterologously expressed P2X_2_Rs in hippocampal neurons and evoke membrane potentials of these neurons in a time-dependent manner [62]. DMNPE-caged ATP was also employed to control the activation of exogenous P2X_3_Rs, which allows for assessing the fast activation kinetics of the whole-cell P2X_3_R-current [25]. Further, Fischer et al. found that photolysis of NPE-caged ATP with a 405 nm laser enabled the fast activation of P2Y_1_Rs in mitral cells, thereby resulting in the increased neuronal network activity in the olfactory bulb, which contributed to our understanding of the physiological role of P2Y_1_Rs in the central nervous system [20].

Recently, caged purinergic receptor agonists and antagonists have also been developed, enabling the control of specific purinergic receptor subtypes. For example, Gao and coworkers synthesized MRS2703, a caged form of a potent dual agonist of P2Y_1_Rs and P2Y_12_Rs (2-methylthio-ADP, (2-MeSADP)) [22] (Fig. 1a). It is inactive at both P2Y_1_Rs and P2Y_12_Rs prior to irradiation. However, upon irradiation at 360 nm for 5 s, photo-uncaging MRS2703 in washed human platelets could activate P2Y_1_Rs and P2Y_12_Rs expressed on the surface of platelets and facilitated the platelets aggregation. Another example is the synthesis of caged A_2A_R antagonist MRS7145 [57] (Fig. 1a). In cultured cells transfecting with A_2A_Rs, photo-uncaging MRS7145 with 405 nm light rapidly activated A_2A_Rs and preclude A_2A_Rs agonist-induced cyclic adenosine monophosphate (cAMP) accumulation. Further, after intraperitoneal injection of MRS7145 into mice, irradiation (405 nm) in the dorsal striatum of mice could significantly induce hyperlocomotion and counteracted haloperidol-induced catalepsy and pilocarpine-induced tremor [57]. These two examples also indicated that the photocontrol of purinergic receptors with caged compounds could provide a new strategy for clinical treatment.

Although photolysis of caged compounds has proven useful for controlling purinergic receptors and dissecting the functions of different purinergic receptors, it also has some limitations. First, as the synthesis of caged compounds is usually complex, biologists are restricted to the few caged compounds that are commercially available or they must collaborate with academic laboratories that synthesize caged compounds [17, 18]. Second, it is still unclear whether the by-products (the cleavage product of the photoremovable protecting group) generate unpredictable cellular or extracellular responses. Considering this, it should be confirmed that these by-products are biologically inert and non-toxic before the experiments [34]. Third, the irreversible nature due to light-induced break of chemical bonds becomes the major limitation, for instance, when one seeks to investigate the opening and closing mechanism of P2XRs.

## Photoswitchable compounds

The photolysis of caged compounds is an irreversible process. Photoswitchable compounds, in contrast, can be used to reversibly manipulate a wide range of biological targets, including G protein-coupled receptors, ion channels, transporters, and enzymes [5, 54, 63]. Light induces conformational changes in these photoswitchable compounds and thereby controls targeted receptors in a time-dependent manner.

Photoswitchable compounds have been successfully employed to optically control purinergic receptors. In two independent groups, photoswitchable compounds, named 4,4′-bis(maleimido)azobenzene (BMA) and maleimide ethylene azobenzene trimethyl ammonium (MEA-TMA), have been synthesized and then were covalently tethered into the outer ends of transmembrane helices of the P2X_2_Rs at residue P329C and I328C, respectively [12, 35]. Light-controlled toggling between cis and trans isomers of azobenzene acts to bring the subunits closer or further apart, thus closing or opening the channel. Importantly, they found that rapid opening of P2X_2_R channels allowed permeation of small cations, such as sodium and calcium ions, but not to chloride ions, indicating that tethered photoswitchable compounds did not alter cation selectivity of the P2X_2_R channel [35].

Similarly, photoswitching has also been applied to manipulate P2X_3_Rs and heteromeric P2X_2/3_Rs. In P2X_3_Rs with P320C mutation, after treatment with BMA, the light at 440 nm rapidly evokes desensitizing currents while light at 360 nm switches off these currents (Fig. 1b) [12]. These light-activated currents are like that activated by a maximal concentration of ATP. The heteromeric P2X_2/3_R channels, which is formed by two P2X_3_R[P320C] subunits and one P2X_2_R subunit, also can be opened and closed by light illumination. This finding indicates that conformational change between only two P2X_3_Rs subunits is sufficient for P2X_2/3_R channel opening [12].

A recent study using photoswitchable tweezer to photocontrol P2X_2_R has contributed to our understanding of the gating mechanism (Habermacher et al., 2016). These photoswitchable tweezers hold strong ability to reveal details of how the subunits move to open or close the P2X_2_R channel’s pore, which overcomes the shortcomings of X-ray crystallography. This strategy entailed the use of a synthesized maleimide azobenzene maleimide (MAM), a photoswitchable azobenzene cross-linker carrying two sulfhydryl-reactive maleimides known to cross-link pairs of an engineered cysteine residue. When attached between I328C from one subunit and S345C from another in P2X_2_R, the cis isomer of MAM induced pore opening by a 525 nm light and the trans isomer induced a closing state by a 365 nm light (Fig. 1b). Combining the photoswitching with computational studies, they further found that the extent of the outer pore expansion is significantly reduced compared to the ATP-bound structure, and the inner and outer ends of adjacent pore-lining helices come closer during opening, likely through a hinge-bending motion.

Photoswitchable tweezers also provide useful molecular rulers to probe the permeation mechanism of P2XRs. Harkat et al. synthesized a shorter, however, more rigid photoswitchable tweezer, named MAM-2 [26]. When this tweezer was covalently attached to residues I328 and S345 of P2X_2_R, 365 nm light at these P2X_2_Rs permits the flow of large synthetic cation, N-methyl-D-glucamine (NMDG +), as well as large natural cation, spermidine. As spermidine is known to modulate a number of ion channels, including synaptic N-methyl-D-aspartate (NMDA) receptors [44], the permeability of the P2X_2_Rs for large cations offers new insights into the physiological function of P2X_2_Rs.

These photoswitchable compounds can be successfully employed to manipulate the opening and closing state of P2XRs and help boost our understanding of their permeation and gating mechanisms. This is achieved by the photoconversion of azobenzenes, which can reversibly switch between a cis form and a trans configuration using two different wavelengths of light, classically near-ultraviolet (360–400 nm) and blue-green light (480–550 nm) [54, 63]. However, the toxicity of azobenzenes, which may stem from cleavage into carcinogenic aromatic amines and metabolic oxidation of amine-bearing azobenzenes to toxic species [41, 60], limits its application in vivo. In addition, the complete recovery of conformational change is not possible due to incomplete cis to trans photoisomerization [11, 40]. However, the recent evidence that silver nanowire antennas enhance the conversion efficiency from around 20 to up to 85% [61] may provide a new strategy to increase the yield of cis/trans isomers. Further, it is entirely possible that photoswitchable compounds could be extended to photocontrol other P2XRs since these P2XRs share similar structures and gating mechanisms.

## Optogenetics for controlling purinergic receptors signaling

Although optopharmacological strategies have proven useful to control purinergic receptors, they have inherent limitations to be used in vivo. Optogenetics overcomes these limitations and has been successfully utilized in vivo. Furthermore, it also enables to control purinergic receptors signaling with spatial and temporal precision, which permits to investigate the behavioral responses upon the control of purinergic receptors signaling.

With the technical advance in opto-A_2A_R and transgenic strategy, optogenetics has been successfully applied to activate or inhibit A_2A_R signaling by light. The opto-A_2A_R is synthesized by retaining the extracellular and transmembrane domains of rhodopsin (conferring light sensitivity and eliminating the binding pockets of adenosine) and replacing the intracellular domain of rhodopsin with that of the A_2A_R (conferring specific A_2A_R signaling) [37]. When opto-A_2A_R is cloned into a viral vector carrying with cell-type-specific promoter, it can be typically introduced into specific subtype neurons in the targeted brain area by stereotaxic microinjection. After 2–3 weeks for the expression of opto-A_2A_R construct in the brain, 473 nm laser light could activate opto-A_2A_R and recruit A_2A_R signaling. As for transgenic strategy, A_2A_R-cre mice, in which the expression of cre recombinase is under the control of A_2A_R gene regulatory elements, are constructed. The use of a cre-dependent viral vector carrying ChR2 into A_2A_R-cre mice is capable of activating A_2A_R signaling by 473 nm light, while the application of a cre-dependent viral vector transforming NpHR into A_2A_R-cre mice enables inhibiting A_2A_R signaling by 593 nm light [28] (Fig. 1c). With these strategies, Oishi and coworkers found that photoactivation of A_2A_R signaling in the core region of the nucleus accumbens of A_2A_R-cre mice induced slow-wave sleep, while such a reaction did not occur when photoactivation was targeted to the shell region of the nucleus accumbens [48]. Hong et al. showed that optogenetic activation of A_2A_R-containing indirect medium spiny projection neurons in the dorsomedial striatum of A_2A_R-cre mice reduced ethanol-containing reward-seeking behavior, whereas optogenetic inhibition of these A_2A_Rs neurons reversed this behavior [28]. Similarly, optogenetic activation of A_2A_R signaling in the dorsomedial striatum selectively impairs the maintenance and retrieval of spatial working memory, but optogenetic activation of A_2A_R signaling in the media prefrontal cortex improves memory maintenance [38]. In addition, optogenetics has also been used to manipulate A_2A_Rs signaling in the hippocampus and striatopallidal pathway, revealing their role in memory and instrumental learning, respectively [27, 37].

Optogenetics has also been utilized to photocontrol P2Y_1_Rs in the vagal nerve. For remote control of P2Y_1_R neurons in the vagal nerve, transgenic P2Y_1_R-ChR2 mice are generated by crossing P2Y_1_R-cre mice with reporter mice containing a cre-dependent ChR2 allele. Focal illumination (473 nm laser) of the nerve trunk or particular nerve branches of P2Y_1_R-ChR2 mice traps breathing in exhalation and does not impact heart rate (Fig. 1c) [14]. Further, Prescott et al. find that vagal P2Y_1_R neurons also engage in an airway defense program. They show that photostimulation (473 nm laser) of P2Y_1_R expressing neurons in the vagal nerve of P2Y_1_R-ChR2 mice evokes a suite of protective reflexes, including apnea, vocal fold adduction, swallowing, and expiratory reflexes [53]. These outcomes suggest that optogenetics also enables a spatial and temporal control of purinergic signaling in peripheral nervous systems.

Clearly, optogenetics is an effective and meaningful tool to control purinergic receptors signaling both in central and peripheral nervous systems. But there are still some issues that are worth mentioning. For instance, it was demonstrated that the introduction of a viral vector for opsin expression could influence the transduction efficiency, tropism, and axonal transport in targeted areas [4, 51]. Meanwhile, opsins in the cells themselves may produce the potential immune response and cause the death of cells [42]. Particularly, surgical implantation of an optical fiber to deliver light to the targeted area produces tissue damage, which limits the application of optogenetics to study large-scale neural networks distributed to different parts of the brain. Although wireless optical equipment has provided an alternative solution [3, 45], optogenetics with low immune response and less invasiveness will allow further control of purinergic signaling and investigate their roles in physiological and pathological conditions.

## Conclusion

As documented above, currently, two distinct types of optical approaches afford powerful and precise manipulation of purinergic signaling (Fig. 1): optopharmacology, which relies on the synthesis of photosensitive chemicals (including caged and photoswitchable compounds), and optogenetics, which requires the genetic modification of the purinergic receptors. With the use of light, these methods enable fast and precise control of targeted purinergic receptors, such as P2X_2_Rs, P2X_3_Rs, P2X_2/3_Rs P2Y_1_Rs, P2Y_12_Rs, and A_2A_Rs. They are also employed to explore the permeation and gating mechanisms of P2XRs, and the role of adenosine receptors in distinct brain areas. They also offer the potential for defining pharmacological targets more precisely.

Although all three optical strategies have been proved powerful and helpful, there are still some problems that have to be solved. Firstly, the delivery of light to the region of interest often requires invasive surgery. Secondly, long-term light stimulation generates heat that leads to permanent tissue damage and affects cellular excitability [49, 52]. In view of that, we suggest the following two considerations when designing experiments: minimization of light power and duration and carefully planned control experiments that account for off-target effects of light delivery. Further, recent advances in magnetogenetics [46] and ultra-sensitive step-function opsin [24], which provide a minimally invasive approach to precisely manipulate neuronal activity in living animals, may overcome these limitations. Ultimately, we are convinced that the elucidation of physiological function and therapeutic potential of purinergic signaling will be further advanced with the development of more intricate and subtle optical tools.

## Abbreviations

ATP: Adenosine triphosphate
Rs: Purinergic receptors
TM: Transmembrane
ADP: Adenosine diphosphate
AMP: Adenosine monophosphate
NPE: P3-1-(2-nitro)phenyl–ethyl
DMB: P3-3′,5′-dimethoxybenzoic acid
DMNPE: P3-[1-(4,5-dimethoxy-2-nitrophenyl)ethyl]
DMACM: 7-(Dimethylamino)coumarin-4-yl]methyl
MRS2703: P1-2(methylthio)adenosyl-P2-(RS)-1-(4,5-dimethoxy-2-nitrophenyl)ethyl pyrophosphate
2-MeSADP: 2-Methylthio-ADP
MRS7145: (7-(Diethylamino)-2-oxo-2H-chromen-4-yl)methyl (2-(furan-2-yl)-7-(3-(4-methoxyphenyl)-propyl)-7H-pyrazolo[4,3-e][1,2,4]triazolo[1,5-c]pyrimidin-5-yl)carbamate
SCH442416: 2-(2-Furanyl)-7-[3-(4-methoxyphenyl)propyl]-7H-pyrazolo[4,3-e][1,2,4]triazolo[1,5-c]p-yrimidin-5-amine
cAMP: Cyclic adenosine monophosphate
BMA: 4,4′**-**Bis(maleimido)azobenzene
MEA-TMA: Maleimide ethylene azobenzene trimethyl ammonium
MAM: Maleimide azobenzene maleimide
NMDG^+^: N-methyl-D-glucamine
ChR2: Channelrhodopsin-2
NpHR: Halorhodopsin
